# OPENING THE DOOR TO AGING IN PLACE: FINDINGS FROM THE INTERGENERATIONAL TORONTO HOMESHARE PROGRAM

**DOI:** 10.1093/geroni/igac059.1992

**Published:** 2022-12-20

**Authors:** Jessica Hsieh, Raza Mirza, Jackie Tanner, Amanda Bull, Andrea Gardiola, Carley Moore, Deirdre Kelly-Adams, Christopher Klinger

**Affiliations:** University of Toronto, Toronto, Ontario, Canada; National Initiative for the Care of the Elderly, Toronto, Ontario, Canada; Natioinal Initiative for the Care of the Elderly, Toronto, Ontario, Canada; National Initiative for the Care of the Elderly, Toronto, Ontario, Canada; National Initative for the Care of the Elderly, Toronto, Ontario, Canada; National Initiative for the Care of the Elderly, Toronto, Ontario, Canada; National Initiative for the Care of the Elderly, Toronto, Ontario, Canada; University of Toronto, Toronto, Ontario, Canada

## Abstract

Older adults prefer to live in their own homes for as long as possible — to 'age in place' — but for myriad reasons, may be unable to do so. To address this, a number of housing alternatives have been explored, including homesharing, or homeshare, an exchange-based shared housing approach with the potential to empower older adults to age in place by enabling them to obtain additional income, companionship, and assistance with completing household tasks in exchange for renting out a room in their home.An intergenerational homesharing pilot program in Toronto matched older adults (55+) with postsecondary students. With limited research in the area, a mixed methods research study was embedded within the pilot project with the goals of: 1) conducting a scoping review to map and synthesize the literature related to outcomes of homeshare participation for this population, 2) conducting in-depth interviews with homeshare participants (N=22) to learn about their experiences, and 3) conduct a full evaluation and exit survey to better understand the implications of the project. Results were organized around the following themes: (1) benefits and challenges of participating in homeshare for older adults; (2) intergenerational engagement as social exchange; and (3) the key role of agency facilitation as a determinant of the experience of homesharing for older adults. Results spoke to the unique benefits and challenges of participating in homeshare for this population. Findings were used to derive implications for policy and practice, as well as highlight areas for future research.

